# How the choice of ethnic indicator influences ethnicity-based inequities in maternal health care in four Latin American countries: who is indigenous?

**DOI:** 10.1186/s12939-020-1136-6

**Published:** 2020-03-12

**Authors:** Nancy Armenta-Paulino, Adela Castelló, María Sandín Vázquez, Francisco Bolúmar

**Affiliations:** 1 Public Health Unit, Faculty of Medicine, University of Alcalá, Crtra Madrid-Barcelona Km 33.6, Alcalá de Henares, 28871 Spain; 2 Ciberesp, Madrid, Spain; 3 Department of Epidemiology and Biostatistics. Graduate School of Public Health, City University of New York, 55 W 125th St, New York, 10027 USA

**Keywords:** Health inequalities, Indigenous, Maternal health care, LAC

## Abstract

**Background:**

The current focus on monitoring health inequalities and the complexity around ethnicity requires careful consideration of how ethnic disparities are measured and presented. This paper aims to determine how inequalities in maternal healthcare by ethnicity change according to different criteria used to classify indigenous populations.

**Methods:**

Nationally representative demographic surveys from Bolivia, Guatemala, Mexico, and Peru (2008–2016) were used to explore coverage gaps across maternal health care by ethnicity using different criteria. Women were classified as indigenous through self-identification (SI), spoken indigenous language (SIL), or indigenous household (IH). We compared the gaps through measuring coverage ratios (CR) with adjusted Poisson regression models.

**Results:**

Proportions of indigenous women changed significantly according to the identification criterion (Bolivia:SI-63.1%/SIL-37.7%; Guatemala:SI-49.7%/SIL-28.2%; Peru:SI-34%/SIL-6.3% & Mexico:SI-29.7%/SIL-6.9%). Indigenous in all countries, regardless of their identification, had less coverage. Gaps in care between indigenous and non-indigenous populations changed, for all indicators and countries, depending on the criterion used (e.g., Bolivia CR for contraceptive-use SI = 0.70, SIL = 0.89; Guatemala CR for skilled-birth-attendant SI = 0.77, SIL = 0.59). The heterogeneity persists when the reference groups are modified and compare just to non-indigenous (e.g., Bolivia CR for contraceptive-use under SI = 0.64, SIL = 0.70; Guatemala CR for Skilled-birth-attendant under SI = 0.77, SIL = 0.57).

**Conclusions:**

The indigenous identification criteria could have an impact on the measurement of inequalities in the coverage of maternal health care. Given the complexity and diversity observed, it is not possible to provide a definitive direction on the best way to define indigenous populations to measure inequalities. In practice, the categorization will depend on the information available. Our results call for greater care in the analysis of ethnicity-based inequalities. A greater understanding on how the indigenous are classified when assessing inequalities by ethnicity can help stakeholders to deliver interventions responsive to the needs of these groups.

## Background

With the adoption of the 2030 Agenda for Sustainable Development, governments pledged to ensure “no one will be left behind”, and to continue efforts to reduce maternal mortality and inequalities in maternal health. Sustainable Development Goals (SDGs) have called for the production of quality, accessible, timely, and reliable “data disaggregated by income, gender, age, ethnicity, disability, and other relevant characteristics,” and subsequently, monitoring health inequalities has gained political attention [1, 2]. Therefore, now seems to be the time to assess ethnicity-based differences in maternal health care.

Unlike other dimensions of inequity, measuring ethnic inequalities is particularly difficult. Studying how inequalities in health vary by ethnicity involves dividing the population into appropriate groups, but the main obstacles are the identification of ethnicity in a consistent or standardized way, and the lack of disaggregated data [3, 4]. In general, ethnicity is not defined by fixed or easily measurable characteristics [3, 5]. For indigenous populations in particular, there are four dimensions that should be considered when establishing operational ethnicity criteria: (i) recognition of identity; (ii) common origin (iii) territoriality; and (iv) the linguistic-cultural dimension [3, 6].

However, the principal criteria used to identify indigenous people are their self-identification and their spoken language. From a human rights approach, self-identification has been considered as the primary criterion, but the inclusion of questions to capture it in surveys is recent, and not all countries use it as a quantifier yet. Traditionally, countries have used the criterion of spoken language, which some of them continue to use to show the socioeconomic and health situation of the indigenous people [3, 4, 7].

Some studies have shown that the size of the indigenous population varies significantly depending on the criteria used to identify them. In specific contexts, people would not self-identify as indigenous due to negative prejudices. Moreover, the use of indigenous languages is gradually decreasing among the younger generations and in urban populations, so, under these criteria indigenous population seem to be decreasing or underestimated [3, 4, 8]. Therefore, using both self-identification and language may provide not only a complete picture of the ethnic inequalities but will also avoid undercount of the indigenous population.

Recently, several studies have explored the inequalities in maternal, newborn and child health by ethnicity in some countries of Latin America [9–12]. The criteria used to identify the women as indigenous were self-identification (SI), spoken indigenous languages (SIL), or indigenous household (IH) (if the head of the household speaks an indigenous language or self-identifies). All studies agree with the vulnerability of indigenous women, who have less coverage across the continuum of care from before pregnancy, through pregnancy to childbirth [9–11]. Although the results are not directly comparable, it is possible to observe how the magnitude of the gaps in the coverage level varies according to the criterion used to identify ethnicity.

For the monitoring of inequalities to be meaningful and useful in reducing ethnic gaps, equal attention should be given to how ethnicity is conceptualized and specified, just as to which is the rationale for reporting ethnic differences. These aspects are essential because without accurate and reliable data, there is little capacity to monitor changes in health status, to evaluate access to services and their response to population needs, or to quantify the resources expended on health services and programs.

To fill these gaps, this study aims to determine how ethnic inequalities in maternal healthcare change according to the different criteria used to classify the indigenous populations.

## Methods

### Study design and data sources

**Fig. 1:**
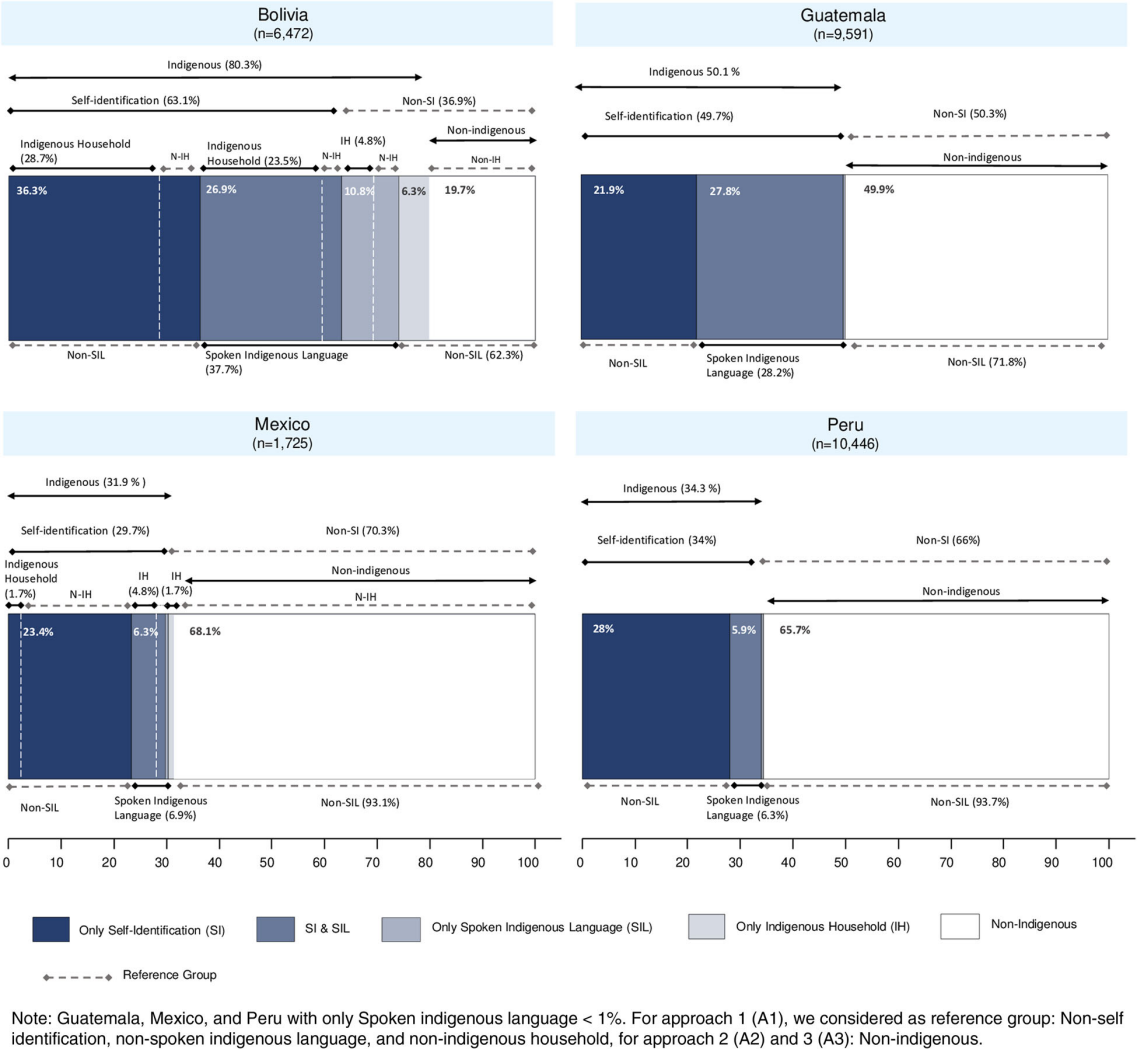
Distribution of each country population according to indigenous criterion

This study is a cross-sectional analysis of the continuum of maternal health care by ethnicity in four Latin American countries: Bolivia, Guatemala, Mexico, and Peru. These countries have a substantial proportion of indigenous population, and a high maternal mortality ratio [13]. The analyses relied on the following publicly available databases: Demographic and Health Surveys and Multiple Indicator Cluster Survey, specifically, Demographic and Health Survey (ENDSA) 2008 (Bolivia), National Survey on Maternal and Child Health (ENSMI) 2014–2015 (Guatemala), National Survey of Children and Women (ENIM) 2015 (Mexico), and Demographic and Family Health Survey (ENDES) 2016 (Peru) [14–16]. All surveys collect individual information on ethnicity and maternal health care. Taken together, the 4 surveys included 28, 240 women. The numbers of women included in the calculation of each indicator are shown in Fig. 1. We used the software Stata 14 for the analysis and graphs.

### Coverage indicators

We assessed six indicators that cover the continuum of care received by women before and during pregnancy as well as childbirth and postpartum: Contraceptive use in women married or living with a partner, antenatal care with a skilled provider, four or more antenatal care visits, first antenatal care visit in the first trimester, skilled birth attendance, and postpartum care (Additional file 1: Table S1). From the perspective of the continuum of care, every phase is essential and reducing mortality depends on both the care coverage and the quality of the services provided [17].

The coverages for each indicator were defined as the percentage of women who receive a specific intervention among those who need it. For indicators related to antenatal care, skilled birth attendance, and postpartum care, women of reproductive age (WRA) of 15–49 years’ old who had delivered a child either in the last 2 (Mexico) or 5 (Bolivia, Guatemala, Peru) years at the time of the survey were included. To analyze the use of contraceptives, we considered WRA currently married or living with a partner.

### Identification of ethnicity

We used the self-identification and language spoken as a proxy for ethnic identity. With respect to self-identification, the surveys inquired if participants belonged to an indigenous group, or if they considered themselves as indigenous or of a specific ethnic group. As regards language, surveys asked whether the woman or the head of the household spoke an indigenous language, what language was spoken at home, or which was the most common language spoken in their home. The questions and criteria used to classify women as indigenous or non-indigenous in each country are summarized in Table 1.

**Table 1:** Survey questions used to ethnic identification

Ethnicity identification Questions		Categorizing	
		Indigenous	Reference Group
*Self- identification (SI)*		SI	Non-SI
Bolivia	Do you consider yourself a member of an indigenous people such as quechua, aymara, guarani, or other?	Quechua, aymara, guaraní, other	None
Guatemala	Do you consider yourself as indigenous or non-indigenous?	Indigenous	Non-indigenous
	Do you consider yourself: maya, ladina/mestiza, garifuna, xinca, or other ethnia?	Maya, xinca or other ethnia	Ladina/mestiza, garifuna
Mexico	According to your culture, do you consider yourself as indigenous?	Yes	No
Peru	According to your ancestors and traditions, do you consider yourself as: Quechua?, Aymara?, Native or Amazonia’s indigenous?, Black, mulatto, afro Peruvian?, White?, Mestizo?, other?	Quechua, aymara, native or Amazonia’s indigenous	Black, mulatto, afro peruvian, White, Mestizo
*Spoken indigenous language (SIL)*		SIL	Non-SIL
Bolivia	What languages do you speak?	Quechua, Aymara, Guaraní, another native	Spanish, foreign language
Guatemala	Usually, what language do you speak at home?	Kaqchiquel, Q’eqchi, K’iche, Mam, Poqomchi’, Tzu’utujil, Q’anjob’al, Ch’orti’, Pocomam, Achi, Akateko, Awakateko, Chalchiteko, Chuj, Itza’, Ixil, Jakatelteko (Popti’), Mopan, Sakapulteko, Sipakapense, Tektiteko, Usapanteko	Spanish
Mexico	Do you speak any indigenous language?	Yes	No
Peru	Usually, what language or dialect do you speak at home?	Quechua, Aymara, another native	Spanish, foreign language
*Indigenous household (IH)*		IH	Non-IH
Bolivia	If the head of household said to speak an indigenous language	Indigenous household: Quechua, Aymara, Guaraní, another native	Non-indigenous household: Spanish, foreign language
Mexico	If the head of household said to speak an indigenous language	Indigenous household: Yes	Non-indigenous household: No

Note: Spanish questions are available in Additional file 1 Table S2

We classified women as indigenous through three main criteria: self- identification (SI), spoken an indigenous language (SIL), belong to an indigenous household (IH, if the head of the household reported speaking an indigenous language). The groups are not mutually exclusive, and thus, some women could be classified as indigenous using different criteria, i.e., a woman who spoke an indigenous language and self-identified as indigenous was included in SIL and SI group. Because of the way the data are collected, it was only possible to use the IH criterion in Bolivia and Mexico. In Guatemala, the couples (husband-wife) dataset could not be generated, and in Peru data about indigenous language was only available for women.

### Statistical analysis

#### Measurement of maternal health care inequalities

We estimated the proportion of indigenous women on the sample by ethnic identification criteria and analyzed the distribution of the continuum of maternal healthcare coverage for each of them. Also, we did an Equiplot to show the level of coverage of each criterion and the distance in coverage between indigenous and non-indigenous people, which represents absolute inequality [18].

We measured a relative inequality by ethnicity through the estimation of adjusted coverage ratios (CR). For each outcome a CR is calculated by dividing the proportion of health care achieved in indigenous versus non-indigenous populations and thus can be interpreted as prevalence ratios (i.e. a CR = 1 means that the coverage is equal between indigenous and non-indigenous). Using a Poisson regression model for outcomes defined as binary variables (Additional file 1: S1 and S3), the adjusted CRs were estimated as a ratio of functions having in the numerator the equation for the category of exposure considered (indigenous) and in the denominator the equation corresponding to the reference group (non-indigenous).

$$ {\boldsymbol{C}\boldsymbol{R}}_{\boldsymbol{X}\mathbf{1}}=\frac{{\boldsymbol{C}}_{\boldsymbol{indigenous}}\ \left({\boldsymbol{X}}_{\mathbf{1}}=\mathbf{1}\right)}{{\boldsymbol{C}}_{\boldsymbol{non}-\boldsymbol{indigenous}}\left({\boldsymbol{X}}_{\mathbf{1}}=\mathbf{0}\right)}=\frac{{\boldsymbol{e}}^{{\boldsymbol{\beta}}_{\mathbf{0}}}{\boldsymbol{e}}^{{\boldsymbol{\beta}}_{\mathbf{1}}\left({\boldsymbol{X}}_{\mathbf{1}}=\mathbf{1}\right)}{\boldsymbol{e}}^{{\boldsymbol{\beta}}_{\mathbf{2}}{\boldsymbol{X}}_{\mathbf{2}}}{\boldsymbol{e}}^{{\boldsymbol{\beta}}_{\mathbf{3}}{\boldsymbol{X}}_{\mathbf{3}}}\dots {\boldsymbol{e}}^{{\boldsymbol{\beta}}_{\boldsymbol{k}}{\boldsymbol{X}}_{\boldsymbol{k}}}}{{\boldsymbol{e}}^{{\boldsymbol{\beta}}_{\mathbf{0}}}{\boldsymbol{e}}^{{\boldsymbol{\beta}}_{\mathbf{1}}\left({\boldsymbol{X}}_{\mathbf{1}}=\mathbf{0}\right)}{\boldsymbol{e}}^{{\boldsymbol{\beta}}_{\mathbf{2}}{\boldsymbol{X}}_{\mathbf{2}}}{\boldsymbol{e}}^{{\boldsymbol{\beta}}_{\mathbf{3}}{\boldsymbol{X}}_{\mathbf{3}}}\dots {\boldsymbol{e}}^{{\boldsymbol{\beta}}_{\boldsymbol{k}}{\boldsymbol{X}}_{\boldsymbol{k}}}}={\boldsymbol{e}}^{{\boldsymbol{\beta}}_{\mathbf{1}}} $$

Where, X_1_ adopts the value of zero “0” in the unexposed group (non-indigenous) and the value of 1 in the exposed group (indigenous).

When the Poisson regression is applied to binomial data, the association between exposure and outcome is directly estimated by means of prevalence ratios (in our case coverage ratios), which are more intuitive and easily interpreted than other association measures such as the odds ratios from logistic regression [19–21].

The Poisson models were adjusted by age, education, socioeconomic level, area of residence, affiliation to some health insurance, and whether women were beneficiary of a social program related to maternal care [3, 4, 22, 23].

#### Differences in the inequalities by ethnic identification criterion used

We compared how ethnic-based inequalities change by indigenous identification criteria through three approaches (Table 2). In the first, we estimated the CR comparing with the direct reference group by each criterion: SI vs Non-SI, SIL vs Non-SIL and IH vs Non-IH. In the second, we considered an adjusted comparison group, i.e., we only included in the reference group women who did not report any of the attributes of ethnic identification: SI vs Non-Indigenous, SIL vs Non-Indigenous and HI vs Non-Indigenous. Finally, in the third, with the same comparison group, we evaluated the CR by ethnicity considering as indigenous women those who reported any of the ethnic identification criteria (SI or SIL or IH).

**Table 2:** Approaches used to analyze ethnic-based inequalities

Approach	Comparison groups		Specifications
A1. Direct references			• The simplest way to measure gaps by ethnicity. We identify as indigenous those who report the specific ethnic criterion and non-indigenous as who do not report it. • Otherwise, if women did not meet these criteria, they were considered as non-indigenous. A woman who self-identified as indigenous but does not speak an indigenous language would be classified as non-indigenous under the SIL criterion. • This could be a unique way to measure inequalities when you only have information on any of the ethnic identification criteria.
	SI vs Non- SI SIL vs Non- SIL IH vs Non- IH		
A2. Adjusted reference groups	SI vs Non- Indigenous SIL vs Non- Indigenous IH vs Non- indigenous		• We only included in the reference group women who did not report any of the attributes of ethnic identification (self-identification, language or living in an indigenous household) • Under this approach, we avoid including in the reference group women with similar sociocultural factors because under other criteria they are identified as indigenous.
A3. Integrate indigenous population	Indigenous if: SI or SIL or IH	vs Non- Indigenous	• We identified as indigenous women those who reported any of the ethnic identification criteria. • Under this strategy, we can identify a higher number of women as indigenous. • We considered that women identified through any of the different criteria could share with the rest some cultural factors related to motherhood or suffer discrimination and have language barriers, all of which may affect maternal health care.

Notes: i) *SI* Self-identification, *SIL* Spoken indigenous language, IH: Indigenous household; ii) To A2 & A3 Non- indigenous are the women who did not report any of the attributes of ethnic identification

We summarized the CRs estimated through these three approaches in a Forest plot to visually inspect the heterogeneity of gaps obtained under different ethnic identification criteria and checked the overlapping of confidence intervals.

## Results

### Indigenous women

There is a certain degree of heterogeneity in the questions used to classify women as indigenous or non-indigenous, with some semantic differences between the countries. Both in the self-identification or language criteria, the questions refer to specific ethnic groups in some cases, and in others, it is asked in a general way (whether it is considered indigenous or not). Some countries ask the question by referring to their culture or with their ancestors (Table 1).

Figure 1 shows that the proportion of indigenous women changes significantly according to the indigenous identification criterion used. In all countries, the percentage of women that self-identify as indigenous (63.2% in Bolivia, 49.7% in Guatemala, 33.9% in Peru and 29.7% in Mexico) is higher than women who said they speak an indigenous language (37.7% in Bolivia, 28.2% in Guatemala, 6.3% in Peru and 6.9% in Mexico). Almost all women in the SIL category are also in the SI category, except in Bolivia where 10.9% of women categorized as SIL are not in the SI category.

### Inequalities in the continuum of care for maternal health

**Fig. 2:**
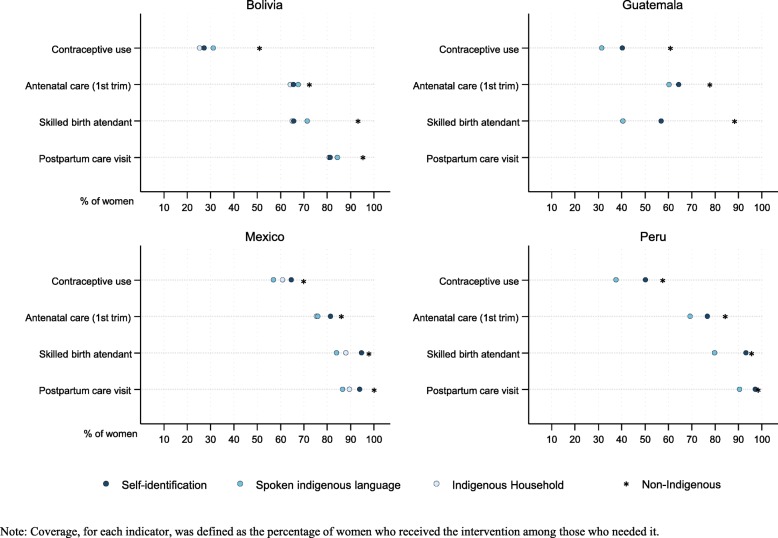
Maternal health care coverage according to indigenous criterion by country

Figure 2 shows the maternal health care coverage for each ethnic criterion and the distance between indigenous and non-indigenous in the four countries. We observed that indigenous women in all four countries, regardless of how they were identified, had less coverage in the continuum of maternal health. Contraceptive use and skilled birth attendant are the components in the continuum of care for maternal health for which the most significant inequalities are observed, mainly for Bolivia and Guatemala. Peru and Mexico appear to be the countries with the smallest gaps throughout the care continuum and Guatemala with the largest care (Additional file 1: Table S4). Regarding socioeconomic characteristics, indigenous women have less education, wealth and access to medical care (Additional file 1: Table S4).

**Fig. 3:**
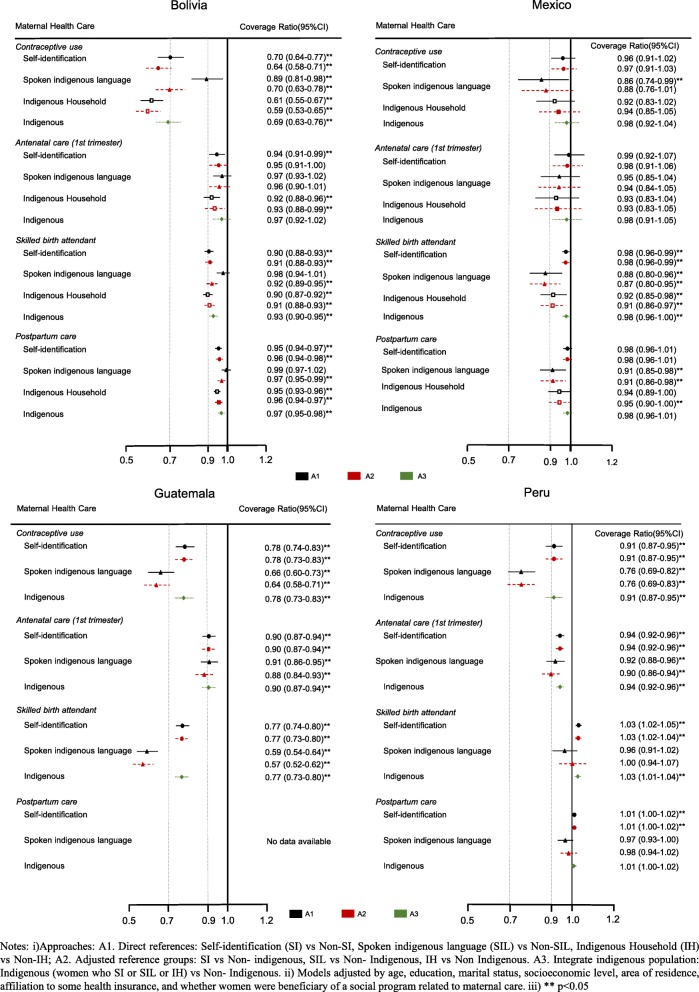
Coverage ratios for the continuum of care for maternal health according to indigenous criterion

These gaps persist after adjusting the CRs by age, education, socioeconomic level, area of residence, affiliation to some health insurance, and whether women were beneficiary of a social program related to maternal care. In Bolivia, Guatemala and Mexico, the contraceptive use and skilled birth attendant have the furthest CR from 1, which means the greater inequalities happen in these phases of the continuum of care, independently of other socioeconomic factors. Also, Guatemala remains the country where the higher inequalities by ethnicity are observed (Tables 3, 4 & Fig. 3).

**Table 3:** Adjusted coverage ratios (95%IC, *p*-value) in indigenous women compared to the reference category, by approach and country (Bolivia & Mexico)

Ethnicity Identification criteria	Approach						
	A1. Direct references			A2. Adjusted reference groups			A3. Integrate indigenous population
Coverage ratio	SI vs Non-SI	SIL vs Non-SIL	IH vs Non-IH	SI vs Non-Indigenous	SIL vs Non-Indigenous	IH vs Non- Indigenous	SI or SIL or IH vs Non-Indigenous
Bolivia							
Before and during pregnancy							
Contraceptive use	0.70 (0.64;0.77) 0.000	0.89 (0.81;0.98) 0.013	0.61 (0.55;0.67) 0.000	0.64 (0.58;0.71) 0.000	0.59 (0.52;0.67) 0.000	0.59 (0.53;0.65) 0.000	0.69 (0.63;0.76) 0.000
Antenatal care (skilled provider)	0.97 (0.95;0.98) 0.000	0.99 (0.98;1.01) 0.531	0.96 (0.95;0.98) 0.000	0.97 (0.95;0.98) 0.000	0.97 (0.95;0.99) 0.001	0.96 (0.95;0.98) 0.000	0.97 (0.96;0.99) 0.000
First antenatal care visit in 1st trimester	0.95 (0.91;0.99) 0.014	0.98 (0.93;1.03) 0.433	0.92 (0.87;0.96) 0.000	0.96 (0.91;1.01) 0.120	0.94 (0.89;1.00) 0.067	0.94 (0.89;0.99) 0.017	0.97 (0.93;1.02) 0.311
Four or more antenatal care visits	0.90 (0.87;0.93) 0.000	1.00 (0.96;1.04) 0.900	0.90 (0.87;0.93) 0.000	0.90 (0.87;0.94) 0.000	0.91 (0.87;0.95) 0.000	0.90 (0.87;0.94) 0.000	0.92 (0.89;0.96) 0.000
Birth and postpartum period							
Skilled birth atendant	0.90 (0.88;0.93) 0.000	0.97 (0.94;1.01) 0.145	0.90 (0.87;0.92) 0.000	0.91 (0.88;0.93) 0.000	0.89 (0.86;0.93) 0.000	0.91 (0.88;0.93) 0.000	0.93 (0.90;0.95) 0.000
Postpartum care visit	0.95 (0.94;0.97) 0.000	0.99 (0.97;1.02) 0.589	0.95 (0.93;0.96) 0.000	0.96 (0.94;0.98) 0.000	0.95 (0.93;0.98) 0.000	0.95 (0.94;0.97) 0.000	0.97 (0.95;0.98) 0.000
Mexico							
Before and during pregnancy							
Contraceptive use	0.96 (0.91;1.02) 0.213	0.86 (0.74;0.99) 0.037	0.92 (0.83;1.02) 0.114	0.97 (0.91;1.03) 0.270	0.88 (0.76;1.01) 0.078	0.94 (0.85;1.05) 0.255	0.98 (0.92;1.04) 0.517
Antenatal care (skilled provider)	0.98 (0.96;1.00) 0.094	0.94 (0.88;1.01) 0.084	0.95 (0.90;1.01) 0.099	0.98 (0.96;1.00) 0.086	0.93 (0.88;1.00) 0.038	0.95 (0.90;1.00) 0.052	0.98 (0.96;1.01) 0.140
First antenatal care visit in 1st trimester	0.99 (0.92;1.07) 0.857	0.95 (0.86;1.05) 0.330	0.93 (0.83;1.05) 0.243	0.99 (0.92;1.06) 0.711	0.95 (0.85;1.06) 0.362	0.94 (0.83;1.06) 0.322	0.98 (0.91;1.06) 0.647
Four or more antenatal care visits	0.98 (0.94;1.01) 0.159	0.85 (0.77;0.95) 0.003	0.89 (0.81;0.96) 0.005	0.97 (0.94;1.00) 0.053	0.85 (0.77;0.93) 0.001	0.88 (0.82;0.96) 0.002	0.97 (0.94;1.00) 0.027
Birth and postpartum period							
Skilled birth atendant	0.98 (0.96;0.99) 0.011	0.88 (0.80;0.96) 0.004	0.92 (0.85;0.98) 0.012	0.98 (0.96;0.99) 0.008	0.87 (0.80;0.95) 0.002	0.91 (0.85;0.97) 0.005	0.98 (0.96;1.00) 0.022
Postpartum care visit	0.98 (0.96;1.01) 0.149	0.91 (0.85;0.98) 0.009	0.95 (0.89;1.00) 0.052	0.98 (0.96;1.01) 0.144	0.91 (0.86;0.98) 0.008	0.95 (0.90;1.00) 0.043	0.98 (0.96;1.01) 0.160

Notes: All models were adjusted by age, education, socioeconomic level, place of residence, marital status, health insurance and social programs related to maternity. SI: Self-identification, SIL: Spoken indigenous language, HI: Indigenous household

**Table 4:** Adjusted coverage ratios (95%IC, *p*-value) in indigenous women compared to the reference category, by approach and country (Guatemala & Peru)

Ethnicity Identification criteria	Approach				
	A1. Direct references		A2. Adjusted reference groups		A3. Integrate indigenous population
Coverage ratio	SI vs Non-SI	SIL vs Non-SIL	SI vs Non-Indigenous	SIL vs Non-Indigenous	SI or SIL or IH vs Non-Indigenous
Guatemala					
Before and during pregnancy					
Contraceptive use	0.78 (0.74;0.83) 0.000	0.66 (0.60;0.73) 0.000	0.78 (0.73;0.83) 0.000	0.64 (0.58;0.71) 0.000	0.78 (0.73;0.83) 0.000
Antenatal care (skilled provider)	0.98 (0.96;1.00) 0.022	0.97 (0.94;1.00) 0.023	0.98 (0.96;1.00) 0.022	0.96 (0.93;0.99) 0.012	0.98 (0.96;1.00) 0.022
First antenatal care visit in 1st trimester	0.91 (0.87;0.94) 0.000	0.91 (0.87;0.96) 0.000	0.91 (0.87;0.94) 0.000	0.89 (0.84;0.93) 0.000	0.91 (0.87;0.94) 0.000
Four or more antenatal care visits	1.00 (0.98;1.02) 0.959	1.02 (0.99;1.05) 0.233	1.00 (0.98;1.02) 0.985	1.02 (0.99;1.05) 0.260	1.00 (0.98;1.02) 0.985
Birth and postpartum period					
Skilled birth atendant	0.77 (0.74;0.80) 0.000	0.59 (0.54;0.64) 0.000	0.77 (0.73;0.80) 0.000	0.57 (0.52;0.62) 0.000	0.76 (0.73;0.80) 0.000
Postpartum care visit					
Peru					
Before and during pregnancy					
Contraceptive use	0.91 (0.87;0.96) 0.000	0.76 (0.70;0.83) 0.000	0.91 (0.87;0.95) 0.000	0.76 (0.69;0.83) 0.000	0.91 (0.87;0.95) 0.000
Antenatal care (skilled provider)	1.00 (1.00;1.01) 0.357	0.94 (0.90;0.98) 0.005	1.00 (0.99;1.01) 0.542	0.96 (0.92;1.00) 0.038	1.00 (0.99;1.01) 0.797
First antenatal care visit in 1st trimester	0.94 (0.92;0.97) 0.000	0.91 (0.87;0.95) 0.000	0.94 (0.92;0.97) 0.000	0.90 (0.86;0.94) 0.000	0.94 (0.92;0.97) 0.000
Four or more antenatal care visits	0.99 (0.99;1.00) 0.284	0.96 (0.94;0.98) 0.001	1.00 (0.99;1.00) 0.331	0.97 (0.95;1.00) 0.025	1.00 (0.99;1.00) 0.309
Birth and postpartum period					
Skilled birth atendant	1.03 (1.02;1.05) 0.000	0.97 (0.91;1.03) 0.331	1.03 (1.01;1.04) 0.000	1.00 (0.94;1.07) 0.990	1.03 (1.01;1.04) 0.000
Postpartum care visit	1.01 (1.00;1.02) 0.016	0.97 (0.93;1.01) 0.117	1.01 (1.00;1.02) 0.029	0.98 (0.94;1.02) 0.407	1.01 (1.00;1.02) 0.049

Notes: All models were adjusted by age, education, socioeconomic level, place of residence, marital status, health insurance and social programs related to maternity. SI: Self-identification, SIL: Spoken indigenous language, IH: Indigenous household

#### Differences among the inequalities by ethnic identification criteria

We observed heterogeneity by the ethnic criterion used for all coverage indicators and countries. The Equiplot (Fig. 2) shows that in almost all countries under the SI criterion the coverage is greater and therefore closer to those of non-indigenous, except for Bolivia, where this happens under the SIL criterion. For example, in Mexico, skilled birth attendance ranges from 84% associated with SIL, 88% with IH, to 95% among SI. The same applies to the sociodemographic characteristics, where some differences by indigenous identification criterion are observed (Additional file 1: Table S4).

Under the first approach (direct references), we observed that among ethnic criteria in Bolivia, when using SIL to identify the women as indigenous and measure the relative inequalities to non-indigenous (non-SIL) the CR is closer to one, i.e., there are fewer inequalities between indigenous and non-indigenous under this criterion. However, if women are identified as indigenous with SI criterion, the CR is far from one, so, under this criterion, the inequalities between indigenous and non-indigenous are greater. For example, the CR for contraceptive use in Bolivia is 30% lower for SI women when compared to non-SI, while there is only an 11% difference between SIL and non SIL women, and the CR is 39% lower among IH women compared to non-IH women (Fig. 3). By contrast, for other countries, the highest CR were observed when the SI criterion was used and was close to 1 in Mexico and Peru. That is, there is a smaller difference in coverage between indigenous and non-indigenous when considered women who are SI and non-SI (Tables 3, 4 & Fig. 3).

For the second approach (adjusted reference groups), the heterogeneity of gaps by indigenous identification criterion persisted. The greatest CRs were observed under SIL criterion for Bolivia and under the SI criterion for Guatemala, México and Perú. We observed, only for Bolivia, that the differences in inequalities between SIL and non SIL decrease with respect to the first approach, suggesting larger gaps with respect to non-indigenous women, especially for contraceptive use (CR from 0.89 to 0.70 using the new reference group) and skilled birth attendant (CR from 0.98 to 0.92) (Tables 3 & 4).

For the rest of the countries, the disparities were also slightly reduced for almost all indicators, specially under the SIL criterion in most cases. In Mexico, except for contraceptive use, under the SI or SIL the CR remained almost the same. In Peru, the CR remained the same under the SI criterion and under SIL criterion the CR for skilled birth attendant increased in such a way that the gap disappeared. In contrast, in Guatemala, the differences in inequalities between the SI and SIL criteria slightly increased for most indicators. Considering the SIL criteria, the differences concerning non-indigenous women are slightly higher (Tables 3, 4 & Fig. 3).

Figure 3 also shows that when considering as indigenous all women who reported SI, SIL, or IH (third approach), the CR are slightly larger and similar to those in the group with higher CRs. In the case of Bolivia, the CRs are like those observed for women who identified as SIL and for the rest of the countries to those of women who identified as SI.

For all countries, we observed overlapping confidence intervals of CRs in almost all indicators to under any criteria or approach. Under the first and second approaches, in Bolivia, Guatemala, and Peru we observed that there is a greater difference between the CRs estimated under SI and SIL criteria and the confidence intervals do not overlap. A similar situation happens in the skilled birth attendant in Guatemala and Mexico (Fig. 3).

## Discussion

Our results show that the choice of the indigenous identification criterion and the reference groups used to estimate inequalities by ethnicity have an impact on the sizes and interpretation of the health care coverage gaps identified between indigenous and non-indigenous populations. The greatest coverage inequalities were observed for indigenous women classified according to the IH criterion in Bolivia and for those classified under the SIL criterion in Mexico, Guatemala and Peru, especially for contraceptive use and, for all except Peru, for skilled birth attendance. The country showing the higher gaps between indigenous and non-indigenous women for these two indicators was Guatemala, closely followed by Bolivia and to a lesser extent by Mexico and Peru.

Measuring inequalities by ethnicity is not an easy task. The main issue is classifying a study population into appropriate groups. Our study, like previous studies [3, 4, 8], also shows that the proportion of indigenous women varies and depends fundamentally on how ethnicity is defined and measured. Unlike other variables, for ethnicity, there is no gold standard on how to define indigenous populations.

From a human rights perspective, self-identification should be considered as the most appropriate criterion [3]. According to our study, this criterion identifies a greater proportion of indigenous women, with the exception of Bolivia, where there are women who, even with other ethnic attributes such as language or living in an indigenous home, do not recognize themselves as indigenous. This finding is consistent with what was recently discussed about the decrease in the proportion of the population that identifies itself as indigenous in Bolivia after the results of the 2012 census [3, 24, 25].

About ethnic inequalities, our results show that, regardless of the criteria used to identify indigenous women, this group of women has lower levels of healthcare coverage before and during pregnancy, childbirth and puerperium compared to other population groups. This is consistent with findings from previous studies and supports estimating inequalities in the continuum of maternal health care disaggregated by ethnicity [9–11].

Our findings also show that contraceptive use and skilled birth attendant are the indicators with the greatest differences by ethnicity, even despite adjustment, which is in alignment with some previous studies [10, 11, 26, 27]. Improving indigenous living conditions could not be enough because other socio-cultural factors are also involved. Usually, indigenous women take care of their pregnancy and childbirth with midwives because of cultural and geographic proximity, they have the recovery of childbirth at home and the decisions about their care involve their partner or another family member [27–31].

The differences identified, with the exception of Bolivia, were most evident when we use the SIL criterion. This finding is consistent with the premise that language is a strong determinant of access to health care [4, 11]. The language is an important element of their cultural attachment [32], so, these women could also have strong rooting for maternal care according to their cultural beliefs. Likewise, the language has been closely linked to the limited access to health care that results from being unable to communicate with health-care personnel [31, 33].

Guatemala is the country with the smaller coverage and the biggest gaps between indigenous and non-indigenous. In this country, the highest maternal mortality and the lower healthcare coverage have been related to socioeconomic factors and weakened national infrastructure derived from a 36-year civil war [27, 34]. However, recently, it has been suggested that conflicting political agendas have contributed to a lack of progress in improving maternal health among indigenous women. The international policies have promoted skilled birth attendance while the domestic policies have sought to strengthen intercultural care provided by traditional birth attendants [34].

Hence, another relevant aspect to consider when discussing our results, is the context in which indigenous populations live in each country. In our study, we observed differences in the socioeconomic characteristics and coverage of care by indigenous identification criterion. The differences observed could be linked to social or cultural factors such as national programs or policies, discrimination, acculturation, maternity habits or gender roles that could affect both the ethnic identification and the use of health services [3, 28, 35–37]. Since knowledge of those factors may be crucial in determining the most appropriate criterion to use as well as an explanation of the inequalities found, more research is required in this regard.

The disparities observed on the proportion of indigenous women identified by the different indigenous identification criterion, translate into differences in the inequalities of health care coverage, that for each country can be larger or smaller depending on how ethnicity is defined.

In view of our results, we consider that when information related to different criteria is available, it would be advisable to analyze the inequalities through each of them in order to determine the most appropriate measure according to the context and objectives in each population. In cases where there are only data based on a single criterion, the potential sensitivity of the possible findings should be recognized. In all cases, researchers or technicians must be explicit about how the indigenous population is defined and categorized, as well as the associated limitations. Reporting these details will be crucial in helping decision makers make informed decisions to reduce the inequalities in maternal health care faced by indigenous women [38–40].

With respect to limitations of our study, we did not use information from the last survey in Bolivia (2016) because its dataset did not provide standardized definitions of the indicators. In addition, the socioeconomic level variable was not available, and this would have prevented adjustment in the models. In Guatemala, we could not use data for postpartum indicators because the sample size would decrease notably due to missing data that was not documented in the survey questionnaire or in the report. Also, we were not able to analyze the temporal trend of the coverages due to the discrepancies over time in the collection of information on the ethnicity of the population within and between countries.

Given the complexity observed in our analysis, it is not possible to provide a definitive direction on the best way to define indigenous populations for the purpose of measuring inequalities. In fact, in practice, the choice of criterion and the categorization of the population will always depend on the information available. However, due to the differences found in the inequalities by indigenous identification criterion, our results call for greater care in the analysis of health-related inequalities by ethnicity and transparency in reporting the measurement approaches taken.

## Supplementary information

**Additional file 1: Table S1.** Definition of indicators. **Table S2.** Spanish survey questions used to ethnic identification. **S3.** Poisson regression model. **Table S4.** Maternal health care coverage and sociodemographic characteristics (95%IC) according indigenous identification criterion by country

## Data Availability

The datasets analyzed during the current study are available in: • The DHS Program: Demographic and Health Surveys (DHS); Available at: https://dhsprogram.com/What-We-Do/survey-search.cfm?pgtype=main&SrvyTp=country • Multiple indicator cluster surveys (MICS); Available at: http://mics.unicef.org/surveys • National Institute of Statistics, and Informatics in Peru; Available at: http://iinei.inei.gob.pe/microdatos/

## Abbreviations

CR: Coverage ratios
IH: Indigenous household
LAC: Latin America and the Caribbean
SDGs: Sustainable Development Goals
SI: Self-identification as indigenous
SIL: Spoken indigenous language
WRA: Women of reproductive age
