# Supplementary material for: How the choice of ethnic indicator influences ethnicity-based inequities in maternal health care in four Latin American countries: who is indigenous?
Source: Int J Equity Health. 2020 Mar 12;19:31. doi: 10.1186/s12939-020-1136-6 (PMC7069165; doi:10.1186/s12939-020-1136-6)
Supplement: Supplementary file 1 — Additional file 1: Table S1. Definition of indicators. Table S2. Spanish survey questions used to ethnic identification. S3. Poisson regression model. Table S4. Maternal health care coverage and sociodemographic characteristics (95%IC) according indigenous identification criterion by country [file 12939_2020_1136_MOESM1_ESM.pdf]

# Supplementary material


*Table S1. Definition of indicators*.............................................1

*Table S2. Spanish survey questions used to ethnic identification*.........................................................................2

*S3. Poisson regression model* ...................................................3

*Table S4. Maternal health care coverage and sociodemographic characteristics (95%IC) according indigenous identification criterion by country* ........................4

**Table S1. Definition of indicators**

| Indicator | Definition | Binary variable |
|---|---|---|
| Before and during pregnancy | | |
| Contraceptive use in married or living with a partner | Percentage of women aged 15–49 years currently married or in union who are using (or whose partner is using) a modern contraceptive method. | 1: If the woman currently married or in union are using (or whose partner is using) a modern contraceptive method<br>0: if doesn't |
| Antenatal care with a skilled provider | Percentage of women attended at least once during pregnancy by skilled health personnel for reasons related to the pregnancy.<br>Bolivia: Doctor, nurse and auxiliary nurse.<br>Guatemala: Doctor, nurse and skilled midwife<br>Mexico: Doctor, nurse and skilled midwife<br>Peru: Doctor, obstetrician and nurse | 1: If the woman attended at least once during pregnancy by skilled health personnel for reasons related to the pregnancy.<br>0: if doesn't |
| First antenatal care visit in the first trimester | Percentage of women with the first antenatal care visit in the first trimester of pregnancy. | 1: If the woman had the first antenatal care visit in the first trimester of pregnancy<br>0: if doesn't |
| Antenatal care visits | Percentage of women with four or more antenatal care visits. | 1: If the woman had four or more antenatal care visits<br>0: if doesn't |
| Birth and postpartum period | | |
| Skilled birth attendance | Percentage of livebirths attended by skilled health personnel.<br>Bolivia, Guatemala, Mexico: Doctor and nurse<br>Peru: Doctor, obstetrician and nurse | 1: If the livebirths were attended by skilled health personnel<br>0: if doesn't |
| Postpartum care | Percentage of women with postpartum care by any provider or time of occurrence. | 1: If the woman had a postpartum care by any provider or time of occurrence.<br>0: if doesn't |

## Table S2. Spanish survey questions used to ethnic identification

| Ethnicity identification Questions | | Categorizing | |
|---|---|---|---|
| | | Indigenous | Reference Group |
| Self- identification (SI) /*Autoidentificación* | | SI | Non-SI |
| Bolivia | *¿Se considera perteneciente a algún pueblo indígena / originario como quechua, aymara, guarani u otro?* | Quechua, aymara, guaraní, other | Ninguno |
| Guatemala | *Usted, ¿cómo se considera indígena o no indígena*? | Indígena | No indígena |
| | *Usted, ¿cómo se considera maya, ladina/mestiza, garífuna, xinca o de otra etnia*? | Maya, xinca or other ethnia | Ladina/mestiza, garifuna |
| Mexico | *De acuerdo con su cultura, ¿usted se considera indígena?* | Si | No |
| Peru | *Por sus antepasados y de acuerdo a sus costumbres usted se considera: ¿Quechua?, ¿Aymara?, ¿Nativo o Indígena de la Amazonía?, ¿Negro /Mulato /Zambo /Afro Peruano?, ¿Blanco?, ¿Mestizo?, ¿Otro?* | Quechua, aymara, Nativo o Indígena de la Amazonía | Negro, Mulato, Afro Peruano, Blanco, Mestizo |
| Spoken indigenous language (SIL) / *Habla lengua indígena* | | SIL | Non-SIL |
| Bolivia | *¿Qué idiomas o lenguas habla?* | Quechua, Aymara, Guaraní, otro nativo | Castellano, extranjero |
| Guatemala | *Usted habitualmente, ¿qué idioma habla en su casa?* | Kaqchiquel, Q'eqchi, K'iche, Mam, Poqomchi', Tzu'utujil, Q'anjob'al, Ch'orti', Pocomam, Achi, Akateko, Awakateko, Chalchiteko, Chuj, Itza', Ixil, Jakatelteko (Popti'), Mopan, Sakapulteko, Sipakapense, Tektiteko, Usapanteko | Español |
| Mexico | *¿Usted habla alguna lengua indígena?* | Si | No |
| Peru | *¿Qué idioma o dialecto hablan habitualmente en su hogar?* | Quechua, Aymara, otra lengua aborigen | Castellano, idioma extranjero |
| Indigenous household (IH) / *Hogar indígena* | | IH | Non-IH |
| Bolivia | *Si el jefe del hogar reporta hablar una lengua indígena* | Indigenous household: Quechua, Aymara, Guaraní, otro nativo | Non-indigenous household: Castellano, extranjero |
| Mexico | *Si el jefe del hogar reporta hablar una lengua indígena* | Indigenous household: Si | Non-indigenous household: No |

## S3. Poisson regression model

When the Poisson regression is applied to binomial data, the association between exposure and outcome is estimated by means of prevalence ratios (PR). In cross-sectional studies, a value of one can be attributed to each participant's follow-up time, as a strategy to obtain PR point estimates, since there is no real follow-up for the participants in this type of studies. In our study since our outcomes are coverage indicators defined as binary variables, under the same approach, we obtained the coverage ratios.

With a Poisson model, we can predict a coverage based on the presence or absence of exposures. The Poisson model with an independent variable ($X_1$):

$$\boldsymbol{Ln(C) = \beta_0 + \beta_1 X_1}$$

Where Ln (C) is the natural logarithm of the coverage or number of events per unit of observation, $X_1$ adopts the value of zero "0" in the unexposed group (non-indigenous) and the value of 1 in the exposed group (indigenous). With this formula we can estimate the predicted the coverage for each of the exposure groups:

Non- Indigenous coverage: $(\boldsymbol{C_0}) \rightarrow \boldsymbol{Ln(C_0) = \beta_0 + \beta_1 * 0 = \beta_0 \rightarrow C_0 = e^{\beta_0}}$

Indigenous rate: $(\boldsymbol{C_1}) \rightarrow \boldsymbol{Ln(C_1) = \beta_0 + \beta_1 * 1 = \beta_0 + \beta_1 \rightarrow C_0 = e^{(\beta_0 + \beta_1)}}$

The coverage ratio (CR) can be estimated as a ratio of functions having in the numerator the equation for the category of exposure considered and in the denominator the equation corresponding to the reference group (or not exposed). Thus, for example, the CR for a variable $X_1$ ($CR_{X1}$) in multiple models would be estimated as follows:

$$\boldsymbol{RC_{X1} = \frac{C_{indigenous}\,(X_1 = 1)}{C_{non-indigenous}(X_1 = 0)} = \frac{e^{\beta_0} e^{\beta_1 (X_1=1)} e^{\beta_2 X_2} e^{\beta_3 X_3} \ldots e^{\beta_k X_k}}{e^{\beta_0} e^{\beta_1 (X_1=0)} e^{\beta_2 X_2} e^{\beta_3 X_3} \ldots e^{\beta_k X_k}} = e^{\beta_1}}$$

**Table S4. Maternal health care coverage and sociodemographic characteristics (95%IC) according indigenous identification criterion by country**

| **Bolivia** | | | | | | | | |
|---|---|---|---|---|---|---|---|---|
| **Ethnicity Identification criterion (% indigenous women)** | **Self-identification 63.1%** | | **Spoken indigenous language 37.7%** | | **Indigenous Household 63.2%** | | **Spoken or Self-identification or Indigenous Household 80.25 %** | |
| **Coverage maternal healthcare** | **SI** | **Non-SI** | **SIL** | **Non-SIL** | **IH** | **Non-IH** | **Indigenous** | **Non-indigenous** |
| Before and during pregnancy | | | | | | | | |
| Contraceptive use | 27.29 (25.83;28.79) | 47.23 (45.09;49.38) | 25.99 (24.11;27.97) | 38.31 (36.72;39.92) | 25.40 (24.08;26.76) | 49.37 (47.28;51.47) | 30.33 (28.96;31.75) | 51.71 (48.82;54.59) |
| Antenatal care (skilled provider) | 86.53 (85.01;87.91) | 96.34 (95.34;97.12) | 87.21 (84.95;89.18) | 91.40 (90.25;92.42) | 86.25 (84.75;87.63) | 96.82 (95.89;97.54) | 88.43 (87.17;89.58) | 97.10 (95.86;97.98) |
| First antenatal care visit in 1st trimester | 65.55 (63.59;67.45) | 73.54 (71.43;75.54) | 65.22 (62.12;68.20) | 70.10 (68.46;71.69) | 64.15 (62.22;66.04) | 75.63 (73.37;77.75) | 67.51 (65.85;69.13) | 73.03 (69.72;76.11) |
| Four or more antenatal care visits | 64.98 (62.82;67.08) | 85.19 (83.44;86.78) | 66.55 (63.37;69.59) | 74.94 (73.16;76.64) | 64.42 (62.29;66.51) | 86.17 (84.46;87.72) | 69.01 (67.19;70.78) | 86.31 (83.82;88.47) |
| Birth and postpartum period | | | | | | | | |
| Skilled birth attendant | 65.58 (63.47;67.64) | 91.22 (89.67;92.55) | 65.79 (62.51;68.93) | 78.98 (77.25;80.61) | 64.95 (62.75;67.10) | 92.35 (90.84;93.63) | 70.82 (68.93;72.64) | 92.14 (90.09;93.79) |
| Postpartum care | 81.26 (79.54;82.87) | 94.37 (93.20;95.34) | 81.29 (78.53;83.77) | 88.14 (86.83;89.34) | 80.85 (79.12;82.47) | 95.09 (93.98;96.00) | 84.03 (82.56;85.40) | 94.48 (92.79;95.78) |
| **Sociodemographic characteristics** | | | | | | | | |
| Age | | | | | | | | |
| 15-19 | 6.64 (5.76;7.63) | 9.69 (8.46;11.08) | 7.20 (5.91;8.73) | 8.00 (7.16;8.93) | 7.23 (6.38;8.19) | 8.67 (7.49;10.03) | 6.92 (6.17;7.76) | 11.17 (9.30;13.37) |
| 20-24 | 18.83 (17.44;20.30) | 25.73 (23.77;27.79) | 20.91 (18.87;23.11) | 21.57 (20.23;22.97) | 20.74 (19.28;22.27) | 22.46 (20.62;24.42) | 20.18 (18.93;21.49) | 26.20 (23.44;29.16) |
| 25-29 | 25.62 (24.01;27.30) | 26.48 (24.22;28.87) | 23.80 (21.55;26.22) | 26.85 (25.23;28.55) | 25.17 (23.64;26.76) | 27.26 (25.00;29.65) | 25.94 (24.55;27.37) | 25.95 (22.83;29.34) |
| 30-34 | 20.70 (19.23;22.25) | 19.39 (17.60;21.32) | 20.53 (18.47;22.75) | 20.08 (18.67;21.57) | 19.56 (18.13;21.08) | 21.34 (19.35;23.48) | 20.45 (19.11;21.86) | 19.26 (16.79;22.01) |
| 35-39 | 15.99 (14.59;17.49) | 12.63 (11.07;14.38) | 16.30 (14.37;18.45) | 14.09 (12.91;15.35) | 15.10 (13.73;16.58) | 14.15 (12.44;16.06) | 15.51 (14.28;16.83) | 11.68 (9.76;13.90) |
| 40-44 | 9.26 (8.24;10.39) | 4.80 (3.91;5.89) | 8.66 (7.18;10.41) | 7.17 (6.32;8.12) | 9.10 (8.07;10.26) | 5.06 (4.12;6.21) | 8.30 (7.41;9.28) | 4.85 (3.72;6.31) |
| 45-49 | 2.97 (2.42;3.63) | 1.27 (0.89;1.83) | 2.60 (1.87;3.59) | 2.23 (1.80;2.77) | 3.09 (2.53;3.78) | 1.05 (0.72;1.54) | 2.70 (2.24;3.26) | 0.88 (0.55;1.41) |
| Education level | | | | | | | | |
| No education | 8.11 (7.06;9.30) | 1.56 (1.10;2.19) | 6.67 (5.45;8.13) | 5.28 (4.46;6.24) | 8.51 (7.44;9.71) | 0.87 (0.59;1.26) | 6.89 (6.02;7.87) | 0.84 (0.53;1.33) |
| Primary | 56.99 (54.94;59.02) | 38.17 (35.84;40.56) | 61.61 (58.74;64.40) | 45.12 (43.19;47.05) | 59.29 (57.35;61.20) | 34.21 (31.86;36.63) | 53.36 (51.54;55.16) | 36.66 (33.55;39.88) |
| Secondary | 25.59 (23.92;27.34) | 40.47 (38.10;42.89) | 24.14 (21.62;26.85) | 34.04 (32.27;35.87) | 24.29 (22.67;26.00) | 42.72 (40.33;45.15) | 28.37 (26.84;29.96) | 42.07 (38.78;45.43) |
| Higher | 9.31 (8.32;10.40) | 19.80 (17.69;22.08) | 7.59 (6.27;9.15) | 15.56 (14.26;16.96) | 7.91 (7.03;8.90) | 22.20 (19.92;24.67) | 11.39 (10.39;12.46) | 20.43 (17.57;23.63) |
| Wealth | | | | | | | | |
| Q1 (poorest) | 30.07 (27.77;32.47) | 9.99 (8.64;11.51) | 31.68 (28.02;35.58) | 18.81 (17.27;20.46) | 30.70 (28.35;33.14) | 8.88 (7.59;10.36) | 25.63 (23.65;27.72) | 10.62 (8.93;12.58) |
| Q2 | 22.81 (21.06;24.66) | 14.61 (12.73;16.72) | 23.71 (20.96;26.71) | 18.11 (16.66;19.65) | 23.78 (21.98;25.67) | 12.94 (11.26;14.83) | 20.91 (19.38;22.52) | 15.25 (12.69;18.22) |
| Q3 | 22.26 (20.41;24.23) | 22.89 (20.76;25.17) | 20.83 (18.33;23.58) | 23.21 (21.62;24.87) | 22.93 (21.10;24.87) | 21.74 (19.74;23.89) | 22.94 (21.34;24.63) | 20.67 (17.91;23.74) |
| Q4 | 16.45 (15.03;17.98) | 25.82 (23.53;28.26) | 15.46 (13.17;18.06) | 21.81 (20.30;23.40) | 14.96 (13.59;16.43) | 28.40 (26.05;30.89) | 18.42 (17.07;19.86) | 25.94 (22.73;29.42) |
| Q5 (richest) | 8.41 (7.40;9.53) | 26.69 (24.25;29.28) | 8.32 (6.81;10.13) | 18.06 (16.53;19.70) | 7.63 (6.67;8.72) | 28.03 (25.47;30.75) | 12.10 (10.96;13.33) | 27.52 (23.98;31.38) |
| Place of residence | | | | | | | | |
| Urban | 46.82 (44.15;49.50) | 76.45 (73.89;78.82) | 43.74 (39.58;47.99) | 63.72 (61.39;66.00) | 44.50 (41.80;47.23) | 80.46 (78.16;82.58) | 52.71 (50.23;55.18) | 78.16 (75.03;80.99) |
| Rural | 53.18 (50.50;55.85) | 23.55 (21.18;26.11) | 56.26 (52.01;60.42) | 36.28 (34.00;38.61) | 55.50 (52.77;58.20) | 19.54 (17.42;21.84) | 47.29 (44.82;49.77) | 21.84 (19.01;24.97) |
| Marital status | | | | | | | | |
| No married | 13.17 (11.98;14.46) | 17.83 (15.97;19.85) | 10.43 (8.76;12.38) | 16.79 (15.49;18.18) | 14.47 (13.20;15.83) | 15.60 (13.82;17.57) | 13.78 (12.70;14.94) | 19.38 (16.51;22.61) |
| Married or union | 86.83 (85.54;88.02) | 82.17 (80.15;84.03) | 89.57 (87.62;91.24) | 83.21 (81.82;84.51) | 85.53 (84.17;86.80) | 84.40 (82.43;86.18) | 86.22 (85.06;87.30) | 80.62 (77.39;83.49) |
| Health insurance | | | | | | | | |
| No | 85.61 (84.24;86.88) | 73.85 (71.45;76.11) | 86.24 (84.04;88.18) | 79.15 (77.63;80.59) | 86.69 (85.37;87.90) | 71.99 (69.71;74.16) | 83.82 (82.55;85.01) | 70.94 (67.70;73.98) |
| Yes | 14.39 (13.12;15.76) | 26.15 (23.89;28.55) | 13.76 (11.82;15.96) | 20.85 (19.41;22.37) | 13.31 (12.10;14.63) | 28.01 (25.84;30.29) | 16.18 (14.99;17.45) | 29.06 (26.02;32.30) |
| Social program | | | | | | | | |
| No | 17.42 (15.85;19.11) | 19.84 (17.67;22.20) | 14.41 (12.31;16.81) | 20.00 (18.40;21.71) | 15.18 (13.70;16.79) | 23.10 (20.72;25.66) | 17.83 (16.43;19.34) | 20.38 (17.20;23.98) |
| Yes | 82.58 (80.89;84.15) | 80.16 (77.80;82.33) | 85.59 (83.19;87.69) | 80.00 (78.29;81.60) | 84.82 (83.21;86.30) | 76.90 (74.34;79.28) | 82.17 (80.66;83.57) | 79.62 (76.02;82.80) |

SI: Self-identification, SIL: Spoken indigenous language, IH: Indigenous household

| Guatemala | | | | | | |
|---|---|---|---|---|---|---|
| **Ethnicity Identification criteria (% indigenous women)** | **Self-identification 49.7%** | | **Spoken indigenous language 28.2%** | | **Spoken or Self-identification 50.1%** | |
| **Coverage maternal healthcare** | **SI** | **Non-SI** | **SIL** | **Non-SIL** | **Indigenous** | **Non-indigenous** |
| Before and during pregnancy | | | | | | |
| Contraceptive use | 40.24 (37.97;42.55) | 59.69 (57.77;61.58) | 31.44 (28.66;34.36) | 57.53 (55.86;59.18) | 40.11 (37.85;42.42) | 59.98 (58.06;61.88) |
| Antenatal care (skilled provider) | 88.82 (87.14;90.31) | 93.78 (92.83;94.61) | 86.15 (83.57;88.39) | 93.35 (92.46;94.14) | 88.82 (87.14;90.31) | 93.82 (92.87;94.66) |
| First antenatal care visit in 1st trimester | 64.45 (62.56;66.30) | 76.87 (75.16;78.51) | 60.34 (57.93;62.70) | 74.78 (73.27;76.24) | 64.44 (62.54;66.29) | 76.98 (75.26;78.60) |
| Four or more antenatal care visits | 84.56 (82.99;86.01) | 87.84 (86.52;89.05) | 83.64 (81.48;85.60) | 87.22 (86.04;88.31) | 84.58 (83.02;86.02) | 87.85 (86.52;89.06) |
| Birth and postpartum period | | | | | | |
| Skilled birth attendant | 56.87 (53.88;59.80) | 86.74 (85.05;88.26) | 40.47 (36.88;44.17) | 84.05 (82.49;85.50) | 56.71 (53.71;59.65) | 87.13 (85.46;88.63) |
| **Sociodemographic characteristics** | | | | | | |
| Age | | | | | | |
| 15-19 | 9.78 (8.84;10.80) | 9.68 (8.73;10.72) | 10.95 (9.69;12.36) | 9.25 (8.46;10.10) | 9.80 (8.87;10.82) | 9.65 (8.70;10.70) |
| 20-24 | 25.80 (24.24;27.43) | 27.63 (26.13;29.17) | 24.66 (22.62;26.81) | 27.53 (26.24;28.87) | 25.92 (24.34;27.56) | 27.53 (26.04;29.06) |
| 25-29 | 24.27 (22.94;25.64) | 26.63 (25.23;28.09) | 22.65 (20.94;24.47) | 26.56 (25.34;27.82) | 24.31 (22.98;25.70) | 26.61 (25.19;28.07) |
| 30-34 | 19.31 (18.10;20.58) | 20.46 (19.20;21.78) | 18.97 (17.41;20.64) | 20.25 (19.20;21.34) | 19.24 (18.04;20.51) | 20.54 (19.27;21.86) |
| 35-39 | 13.18 (12.21;14.20) | 9.86 (9.00;10.80) | 14.04 (12.79;15.38) | 10.52 (9.75;11.34) | 13.10 (12.14;14.13) | 9.91 (9.04;10.86) |
| 40-44 | 6.06 (5.36;6.84) | 4.71 (4.03;5.50) | 6.62 (5.70;7.67) | 4.90 (4.34;5.52) | 6.03 (5.33;6.81) | 4.73 (4.04;5.52) |
| 45-49 | 1.60 (1.25;2.05) | 1.03 (0.75;1.42) | 2.11 (1.57;2.83) | 1.00 (0.77;1.30) | 1.59 (1.24;2.04) | 1.04 (0.75;1.43) |
| Education level | | | | | | |
| No education | 24.67 (22.74;26.70) | 9.89 (8.75;11.16) | 33.94 (31.35;36.63) | 10.67 (9.63;11.81) | 24.71 (22.79;26.74) | 9.73 (8.60;10.99) |
| Primary | 54.72 (52.78;56.65) | 48.01 (45.91;50.12) | 56.04 (53.32;58.72) | 49.50 (47.81;51.19) | 54.81 (52.89;56.72) | 47.87 (45.75;50.00) |
| Secondary | 18.75 (17.04;20.59) | 35.77 (33.68;37.92) | 9.76 (8.05;11.80) | 34.21 (32.49;35.96) | 18.63 (16.93;20.46) | 36.02 (33.92;38.18) |
| Higher | 1.86 (1.43;2.42) | 6.33 (5.48;7.30) | 0.26 (0.13;0.54) | 5.62 (4.95;6.38) | 1.85 (1.42;2.40) | 6.38 (5.52;7.36) |
| Wealth | | | | | | |
| Q1 (poorest) | 34.74 (31.78;37.83) | 13.58 (11.94;15.41) | 50.65 (46.71;54.59) | 13.66 (12.18;15.29) | 34.86 (31.90;37.95) | 13.30 (11.66;15.14) |
| Q2 | 25.30 (23.29;27.42) | 17.53 (16.05;19.12) | 29.07 (26.47;31.81) | 18.37 (17.02;19.81) | 25.27 (23.27;27.40) | 17.49 (16.00;19.10) |
| Q3 | 19.02 (17.25;20.92) | 21.29 (19.70;22.98) | 14.40 (12.23;16.89) | 22.42 (20.94;23.98) | 19.05 (17.26;20.97) | 21.28 (19.68;22.97) |
| Q4 | 13.17 (11.64;14.86) | 24.83 (23.01;26.75) | 4.66 (3.70;5.86) | 24.68 (23.12;26.31) | 13.08 (11.57;14.77) | 25.00 (23.17;26.93) |
| Q5 (richest) | 7.78 (6.57;9.18) | 22.76 (20.86;24.78) | 1.21 (0.78;1.88) | 20.86 (19.19;22.63) | 7.73 (6.53;9.13) | 22.92 (21.01;24.96) |
| Place of residence | | | | | | |
| Urban | 29.83 (26.80;33.05) | 45.92 (43.09;48.77) | 18.45 (14.58;23.08) | 45.57 (43.09;48.07) | 29.67 (26.66;32.88) | 46.20 (43.35;49.07) |
| Rural | 70.17 (66.95;73.20) | 54.08 (51.23;56.91) | 81.55 (76.92;85.42) | 54.43 (51.93;56.91) | 70.33 (67.12;73.34) | 53.80 (50.93;56.65) |
| Marital status | | | | | | |
| No married | 11.59 (10.54;12.73) | 16.44 (15.23;17.72) | 9.14 (7.81;10.67) | 15.95 (14.94;17.02) | 11.58 (10.54;12.71) | 16.49 (15.27;17.77) |
| Married or union | 88.41 (87.27;89.46) | 83.56 (82.28;84.77) | 90.86 (89.33;92.19) | 84.05 (82.98;85.06) | 88.42 (87.29;89.46) | 83.51 (82.23;84.73) |
| Health insurance | | | | | | |
| No | 92.98 (91.89;93.93) | 81.55 (79.73;83.25) | 97.81 (97.02;98.39) | 83.08 (81.64;84.43) | 93.03 (91.95;93.98) | 81.42 (79.58;83.12) |
| Yes | 7.02 (6.07;8.11) | 18.45 (16.75;20.27) | 2.19 (1.61;2.98) | 16.92 (15.57;18.36) | 6.97 (6.02;8.05) | 18.58 (16.88;20.42) |

SI: Self-identification, SIL: Spoken indigenous language, IH: Indigenous household

| Mexico | | | | | | | | |
|---|---|---|---|---|---|---|---|---|
| **Ethnicity Identification criteria (% indigenous women)** | **Self-identification 29.7%** | | **Spoken indigenous language 6.9%** | | **Indigenous Household 8.5%** | | **Spoken or Self-identification or Indigenous Household 31.9%** | |
| **Coverage maternal healthcare** | **SI** | **Non-SI** | **SIL** | **Non-SIL** | **IH** | **Non-IH** | **Indigenous** | **Non-indigenous** |
| Before and during pregnancy | | | | | | | | |
| Contraceptive use | 64.61 (61.06;68.01) | 69.22 (65.98;72.27) | 57.03 (48.82;64.88) | 68.72 (65.97;71.35) | 60.90 (54.47;66.97) | 68.55 (65.76;71.21) | 65.48 (61.93;68.87) | 68.98 (65.67;72.11) |
| Antenatal care (skilled provider) | 96.32 (92.68;98.19) | 99.06 (98.53;99.40) | 91.41 (81.66;96.22) | 98.65 (97.66;99.23) | 92.97 (84.73;96.93) | 98.64 (97.63;99.22) | 96.43 (93.07;98.19) | 98.97 (98.25;99.39) |
| First antenatal care visit in 1st trimester | 81.33 (76.31;85.48) | 84.49 (81.19;87.29) | 75.93 (68.56;82.03) | 84.06 (81.20;86.56) | 75.49 (67.06;82.33) | 84.26 (81.30;86.83) | 80.85 (76.19;84.79) | 84.79 (81.23;87.77) |
| Four or more antenatal care visits | 91.38 (87.61;94.09) | 95.72 (94.16;96.88) | 78.80 (68.54;86.39) | 95.45 (94.04;96.55) | 82.28 (73.25;88.73) | 95.44 (94.06;96.51) | 90.81 (87.29;93.42) | 95.96 (94.53;97.03) |
| Birth and postpartum period | | | | | | | | |
| Skilled birth attendant | 94.72 (91.65;96.70) | 99.09 (98.62;99.40) | 84.00 (74.69;90.33) | 98.74 (97.82;99.28) | 87.92 (79.77;93.07) | 98.65 (97.70;99.21) | 94.94 (92.05;96.82) | 99.04 (98.46;99.41) |
| Postpartum care | 93.85 (91.20;95.74) | 96.74 (95.53;97.63) | 86.51 (78.53;91.83) | 96.59 (95.57;97.38) | 89.56 (82.24;94.08) | 96.49 (95.45;97.30) | 93.98 (91.33;95.86) | 96.79 (95.62;97.66) |
| **Sociodemographic characteristics** | | | | | | | | |
| Age | | | | | | | | |
| 15-19 | 12.41 (9.25;16.43) | 13.58 (11.18;16.39) | 9.25 (6.31;13.37) | 13.52 (11.47;15.86) | 11.17 (7.77;15.81) | 13.41 (11.34;15.79) | 12.50 (9.48;16.31) | 13.56 (11.11;16.45) |
| 20-24 | 32.34 (27.70;37.35) | 33.71 (29.43;38.29) | 30.28 (23.18;38.46) | 33.48 (29.83;37.35) | 36.68 (25.89;49.00) | 32.94 (29.41;36.66) | 34.14 (28.76;39.97) | 32.84 (28.68;37.28) |
| 25-29 | 24.98 (20.70;29.82) | 23.10 (20.06;26.45) | 19.87 (14.55;26.54) | 23.94 (21.31;26.78) | 19.20 (13.92;25.89) | 24.07 (21.41;26.94) | 24.31 (20.10;29.07) | 23.35 (20.27;26.74) |
| 30-34 | 16.89 (13.59;20.80) | 16.48 (13.98;19.32) | 23.34 (16.33;32.21) | 16.06 (14.08;18.26) | 14.97 (10.24;21.35) | 16.72 (14.64;19.04) | 16.09 (12.93;19.86) | 16.80 (14.25;19.70) |
| 35-39 | 8.34 (6.07;11.35) | 9.58 (6.63;13.65) | 8.59 (4.97;14.46) | 9.32 (6.94;12.42) | 11.12 (6.30;18.88) | 9.10 (6.72;12.21) | 8.27 (6.08;11.15) | 9.74 (6.73;13.90) |
| 40-44 | 4.23 (2.42;7.28) | 3.47 (2.16;5.52) | 7.51 (3.99;13.71) | 3.45 (2.29;5.16) | 5.92 (2.97;11.43) | 3.52 (2.35;5.26) | 3.92 (2.24;6.78) | 3.63 (2.29;5.73) |
| 45-49 | 0.82 (0.32;2.05) | 0.07 (0.02;0.24) | 1.15 (0.43;3.02) | 0.23 (0.08;0.63) | 0.94 (0.35;2.50) | 0.23 (0.09;0.64) | 0.76 (0.30;1.91) | 0.08 (0.02;0.25) |
| Education level | | | | | | | | |
| No education | 2.83 (1.69;4.71) | 0.99 (0.63;1.56) | 5.66 (3.03;10.36) | 1.30 (0.84;1.99) | 5.31 (2.88;9.62) | 1.25 (0.80;1.94) | 2.86 (1.74;4.66) | 1.01 (0.58;1.73) |
| Primary | 25.44 (20.88;30.61) | 13.33 (10.80;16.33) | 44.17 (35.90;52.76) | 14.88 (12.54;17.56) | 33.42 (24.14;44.20) | 15.34 (12.92;18.12) | 24.98 (20.46;30.12) | 13.09 (10.54;16.13) |
| Secondary | 43.46 (38.10;48.97) | 40.42 (36.19;44.79) | 35.20 (27.66;43.56) | 41.83 (38.20;45.54) | 44.19 (32.86;56.16) | 41.12 (37.53;44.81) | 43.83 (38.28;49.54) | 40.24 (36.04;44.59) |
| Higher | 28.27 (23.45;33.65) | 45.27 (40.90;49.71) | 14.97 (8.61;24.75) | 41.99 (38.16;45.92) | 17.07 (11.65;24.31) | 42.29 (38.44;46.23) | 28.33 (23.59;33.60) | 45.67 (41.32;50.08) |
| Wealth | | | | | | | | |
| Q1 (poorest) | 45.80 (40.18;51.52) | 14.85 (12.35;17.77) | 70.48 (59.22;79.70) | 20.69 (17.77;23.95) | 62.04 (47.56;74.65) | 20.58 (17.65;23.85) | 44.34 (38.54;50.29) | 14.64 (12.02;17.71) |
| Q2 | 25.93 (21.57;30.82) | 29.48 (25.67;33.60) | 19.38 (11.94;29.88) | 29.09 (25.89;32.51) | 18.43 (12.36;26.58) | 29.35 (26.10;32.82) | 25.83 (21.67;30.49) | 29.62 (25.66;33.92) |
| Q3 | 15.34 (11.70;19.86) | 23.47 (19.74;27.66) | 5.50 (2.99;9.89) | 22.23 (19.12;25.69) | 15.63 (5.75;36.00) | 21.59 (18.58;24.94) | 16.79 (12.21;22.64) | 23.10 (19.41;27.24) |
| Q4 | 8.64 (6.06;12.18) | 18.71 (15.87;21.92) | 3.36 (1.45;7.59) | 16.56 (14.29;19.11) | 3.65 (1.84;7.10) | 16.77 (14.48;19.35) | 8.87 (6.41;12.14) | 18.83 (15.95;22.11) |
| Q5 (richest) | 4.30 (2.92;6.29) | 13.49 (10.30;17.47) | 1.28 (0.49;3.31) | 11.43 (8.98;14.44) | 0.25 (0.03;1.77) | 11.71 (9.22;14.77) | 4.18 (2.87;6.05) | 13.81 (10.55;17.86) |
| Place of residence | | | | | | | | |
| Urban | 59.05 (53.07;64.77) | 81.54 (77.17;85.23) | 43.47 (30.01;57.96) | 77.11 (72.71;80.98) | 50.19 (36.35;63.99) | 77.08 (72.66;80.97) | 60.53 (54.42;66.32) | 81.47 (76.98;85.24) |
| Rural | 40.95 (35.23;46.93) | 18.46 (14.77;22.83) | 56.53 (42.04;69.99) | 22.89 (19.02;27.29) | 49.81 (36.01;63.65) | 22.92 (19.03;27.34) | 39.47 (33.68;45.58) | 18.53 (14.76;23.02) |
| Marital status | | | | | | | | |
| No married | 17.01 (13.59;21.08) | 20.28 (17.16;23.80) | 12.57 (7.71;19.84) | 19.83 (17.23;22.72) | 13.77 (8.68;21.15) | 19.85 (17.23;22.75) | 16.81 (13.48;20.77) | 20.51 (17.34;24.08) |
| Married or union | 82.99 (78.92;86.41) | 79.72 (76.20;82.84) | 87.43 (80.16;92.29) | 80.17 (77.28;82.77) | 86.23 (78.85;91.32) | 80.15 (77.25;82.77) | 83.19 (79.23;86.52) | 79.49 (75.92;82.66) |
| Health insurance | | | | | | | | |
| No | 9.76 (6.18;15.08) | 10.67 (8.50;13.31) | 7.33 (4.49;11.73) | 10.65 (8.67;13.02) | 6.59 (3.94;10.82) | 10.78 (8.77;13.19) | 9.44 (6.05;14.45) | 10.88 (8.63;13.63) |
| Yes | 90.24 (84.92;93.82) | 89.33 (86.69;91.50) | 92.67 (88.27;95.51) | 89.35 (86.98;91.33) | 93.41 (89.18;96.06) | 89.22 (86.81;91.23) | 90.56 (85.55;93.95) | 89.12 (86.37;91.37) |
| Social program | | | | | | | | |
| No | 68.18 (63.05;72.91) | 83.22 (80.01;85.99) | 50.19 (39.48;60.88) | 80.84 (77.94;83.43) | 56.85 (45.24;67.76) | 80.76 (77.81;83.40) | 69.44 (64.31;74.13) | 83.07 (79.79;85.91) |
| Yes | 31.82 (27.09;36.95) | 16.78 (14.01;19.99) | 49.81 (39.12;60.52) | 19.16 (16.57;22.06) | 43.15 (32.24;54.76) | 19.24 (16.60;22.19) | 30.56 (25.87;35.69) | 16.93 (14.09;20.21) |

SI: Self-identification, SIL: Spoken indigenous language, IH: Indigenous household

| Peru | | | | | | |
|---|---|---|---|---|---|---|
| **Ethnicity Identification criteria (% indigenous women)** | **Self-identification 34.0%** | | **Spoken indigenous language 6.3%** | | **Spoken or Self-identification 34.3%** | |
| **Coverage maternal healthcare** | **SI** | **Non-SI** | **SIL** | **Non-SIL** | **Indigenous** | **Non-indigenous** |
| Before and during pregnancy | | | | | | |
| Contraceptive use | 50.16 (48.38;51.95) | 56.49 (54.96;58.00) | 37.56 (34.68;40.54) | 55.53 (54.29;56.77) | 50.12 (48.34;51.90) | 56.54 (55.01;58.06) |
| Antenatal care (skilled provider) | 96.97 (96.04;97.68) | 97.86 (97.36;98.26) | 89.36 (84.80;92.68) | 98.10 (97.70;98.43) | 96.78 (95.73;97.58) | 97.96 (97.48;98.35) |
| First antenatal care visit in 1st trimester | 76.62 (75.13;78.04) | 83.55 (82.60;84.46) | 69.35 (66.20;72.32) | 81.98 (81.16;82.77) | 76.60 (75.13;78.01) | 83.60 (82.65;84.50) |
| Four or more antenatal care visits | 95.24 (94.39;95.97) | 96.68 (96.16;97.13) | 90.94 (88.56;92.85) | 96.54 (96.09;96.94) | 95.24 (94.39;95.96) | 96.68 (96.16;97.14) |
| Birth and postpartum period | | | | | | |
| Skilled birth attendant | 93.26 (92.00;94.34) | 94.11 (93.17;94.92) | 79.79 (74.39;84.29) | 94.76 (94.01;95.41) | 92.96 (91.58;94.14) | 94.27 (93.34;95.07) |
| Postpartum care | 97.24 (96.45;97.86) | 97.58 (96.92;98.10) | 90.46 (86.55;93.32) | 97.93 (97.42;98.34) | 97.12 (96.23;97.80) | 97.65 (96.99;98.16) |
| **Sociodemographic characteristics** | | | | | | |
| Age | | | | | | |
| 15-19 | 5.44 (4.81;6.14) | 5.04 (4.53;5.60) | 8.65 (7.11;10.47) | 4.94 (4.54;5.38) | 5.60 (4.96;6.32) | 4.95 (4.45;5.50) |
| 20-24 | 18.89 (17.73;20.10) | 19.07 (18.14;20.04) | 21.70 (19.13;24.50) | 18.83 (18.06;19.62) | 18.94 (17.77;20.16) | 19.04 (18.11;20.01) |
| 25-29 | 23.46 (22.16;24.82) | 23.50 (22.44;24.60) | 20.62 (18.37;23.08) | 23.68 (22.80;24.58) | 23.36 (22.06;24.70) | 23.56 (22.49;24.66) |
| 30-34 | 24.15 (22.69;25.67) | 22.75 (21.66;23.89) | 16.86 (14.73;19.23) | 23.65 (22.72;24.61) | 24.02 (22.57;25.53) | 22.81 (21.71;23.95) |
| 35-39 | 16.19 (15.06;17.39) | 18.26 (17.20;19.37) | 17.10 (15.14;19.26) | 17.59 (16.74;18.47) | 16.23 (15.10;17.42) | 18.25 (17.19;19.36) |
| 40-44 | 9.47 (8.57;10.45) | 9.05 (8.34;9.81) | 11.57 (9.84;13.56) | 9.03 (8.45;9.66) | 9.46 (8.57;10.44) | 9.05 (8.34;9.82) |
| 45-49 | 2.41 (1.98;2.92) | 2.33 (1.95;2.77) | 3.50 (2.46;4.96) | 2.28 (1.97;2.62) | 2.40 (1.97;2.91) | 2.33 (1.95;2.78) |
| Education level | | | | | | |
| No education | 2.51 (2.08;3.03) | 1.03 (0.81;1.31) | 6.76 (5.22;8.71) | 1.19 (0.99;1.41) | 2.60 (2.15;3.13) | 0.98 (0.76;1.25) |
| Primary | 24.20 (22.80;25.66) | 18.64 (17.31;20.04) | 54.34 (51.25;57.40) | 18.27 (17.23;19.36) | 24.39 (22.99;25.86) | 18.50 (17.18;19.91) |
| Secondary | 49.82 (48.06;51.58) | 45.26 (43.75;46.77) | 34.59 (31.48;37.84) | 47.62 (46.35;48.90) | 49.70 (47.93;51.47) | 45.30 (43.79;46.81) |
| Higher | 23.47 (21.88;25.13) | 35.08 (33.41;36.77) | 4.31 (3.08;6.00) | 32.92 (31.57;34.30) | 23.31 (21.73;24.97) | 35.22 (33.55;36.93) |
| Wealth | | | | | | |
| Q1 (poorest) | 28.18 (26.28;30.15) | 16.86 (15.30;18.55) | 76.25 (72.50;79.64) | 17.00 (15.80;18.27) | 28.58 (26.65;30.60) | 16.59 (15.02;18.29) |
| Q2 | 28.22 (26.47;30.03) | 20.86 (19.52;22.27) | 17.67 (14.96;20.76) | 23.74 (22.52;25.01) | 28.01 (26.27;29.82) | 20.93 (19.59;22.34) |
| Q3 | 20.33 (18.83;21.91) | 21.78 (20.53;23.09) | 3.98 (2.48;6.33) | 22.44 (21.37;23.55) | 20.29 (18.81;21.86) | 21.81 (20.56;23.12) |
| Q4 | 14.47 (13.12;15.93) | 20.42 (19.18;21.71) | 1.27 (0.63;2.52) | 19.54 (18.49;20.63) | 14.39 (13.05;15.84) | 20.49 (19.25;21.79) |
| Q5 (richest) | 8.81 (7.54;10.27) | 20.08 (18.37;21.89) | 0.83 (0.28;2.43) | 17.28 (15.87;18.78) | 8.73 (7.47;10.18) | 20.18 (18.47;22.00) |
| Place of residence | | | | | | |
| Urban | 64.02 (61.74;66.23) | 79.67 (77.73;81.48) | 19.38 (15.93;23.36) | 78.02 (76.58;79.39) | 63.68 (61.38;65.91) | 79.93 (77.97;81.75) |
| Rural | 35.98 (33.77;38.26) | 20.33 (18.52;22.27) | 80.62 (76.64;84.07) | 21.98 (20.61;23.42) | 36.32 (34.09;38.62) | 20.07 (18.25;22.03) |
| Marital status | | | | | | |
| No married | 13.01 (12.00;14.10) | 16.70 (15.73;17.71) | 12.76 (10.86;14.95) | 15.62 (14.87;16.41) | 13.09 (12.08;14.18) | 16.67 (15.70;17.69) |
| Married or union | 86.99 (85.90;88.00) | 83.30 (82.29;84.27) | 87.24 (85.05;89.14) | 84.38 (83.59;85.13) | 86.91 (85.82;87.92) | 83.33 (82.31;84.30) |
| Health insurance | | | | | | |
| No | 14.94 (13.77;16.19) | 16.07 (15.07;17.11) | 8.45 (6.41;11.06) | 16.17 (15.35;17.02) | 14.99 (13.81;16.24) | 16.05 (15.05;17.10) |
| Yes | 85.06 (83.81;86.23) | 83.93 (82.89;84.93) | 91.55 (88.94;93.59) | 83.83 (82.98;84.65) | 85.01 (83.76;86.19) | 83.95 (82.90;84.95) |
| Social program | | | | | | |
| No | 81.50 (79.94;82.97) | 89.22 (87.95;90.37) | 51.81 (47.54;56.04) | 88.92 (87.93;89.83) | 81.28 (79.68;82.77) | 89.38 (88.10;90.53) |
| Yes | 18.50 (17.03;20.06) | 10.78 (9.63;12.05) | 48.19 (43.96;52.46) | 11.08 (10.17;12.07) | 18.72 (17.23;20.32) | 10.62 (9.47;11.90) |

SI: Self-identification, SIL: Spoken indigenous language, IH: Indigenous household
